# Fine-tuned regulation of photosynthetic performance via γ-aminobutyric acid (GABA) supply coupled with high initial cell density culture for economic starch production in microalgae

**DOI:** 10.1186/s40643-022-00541-3

**Published:** 2022-05-12

**Authors:** Yunyun Pan, Yuhan Shen, Haoyu Zhang, Xiuyuan Ran, Tonghui Xie, Yongkui Zhang, Changhong Yao

**Affiliations:** Department of Pharmaceutical & Biological Engineering, School of Chemical Engineering, Sichuan University, Chengdu, 610065 Sichuan China

**Keywords:** Microalgae, Starch, High initial cell density, γ-Aminobutyric acid, Photosynthetic performance, Stress

## Abstract

Microalgal starch is considered as renewable and sustainable feedstock for biofuels and biorefinery. High cell density culture is favourable for photoautotrophic starch production in microalgae in the aspects of productivity and economy, but it often encounters low starch content or extra stress exposure that limits the production. This study aimed to economically enhance photosynthetic starch production from CO_2_ fixation in a green microalga *Tetraselmis subcordiformis* by regulating photosynthetic stress status with a signalling molecule γ-aminobutyric acid (GABA) combined with the application of high initial cell density culture. By increasing initial cell density (ICD) from the normal of 1.1 g L^−1^ (NICD) to as high as 2.8 g L^−1^ (HICD), the starch content, yield, and theoretical productivity were improved by 7%, 63%, and 42%, respectively. The addition of GABA under HICD resulted in 14%, 19%, and 26% of further enhancement in starch content, yield, and theoretical productivity, respectively. GABA exhibited distinct regulatory mechanisms on photosynthesis and stress status under HICD relative to NICD. GABA augmented excessive light energy absorption and electron transfer through photosystem II that reinforced the photoinhibition under NICD, while alleviated the stress reversely under HICD, both of which facilitated starch production by enabling a suitable stress status while simultaneously maintaining a sufficient photosynthetic activity. The increase of ICD and/or GABA supply particularly boosted amylopectin accumulation, leading to the changes in starch composition and was more favourable for fermentation-based biofuels production. Preliminary techno-economic analysis showed that the highest net extra benefit of 9.64 $ m^−3^ culture could be obtained under HICD with 2.5 mM GABA supply where high starch content (62%DW) and yield (2.5 g L^−1^) were achieved. The combined HICD-GABA regulation was a promising strategy for economic starch production from CO_2_ by microalgae for sustainable biomanufacturing.

**Graphical Abstract:**

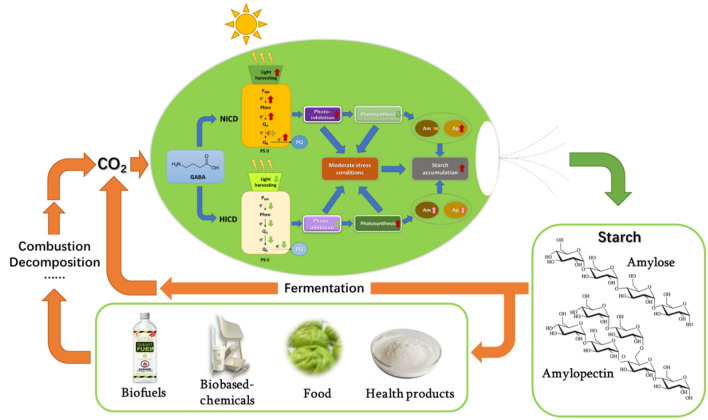

**Supplementary Information:**

The online version contains supplementary material available at 10.1186/s40643-022-00541-3.

## Introduction

Microalgae appear as a kind of photosynthetic organisms that has gained public attention for several decades due to their ability to efficiently sequestrate CO_2_ accompanied with the production of biofuels, such as biodiesel and high-value products, like DHA, polysaccharides, β-carotene, and protein (Allen et al. 2018; Xi et al. 2022; Siddiki et al. 2022). Starch often serves as the primary photosynthetic carbon reserve among many microalgae, especially in the green lineage (Vitova et al. 2015). Starch in microalgae cells exists as similar structures to higher plants starch that can replace crop-based starch for industrial biomanufacturing (Ran et al. 2019). In recent years the growing concern on the global environmental issues highlights the pressing need for a large number of eco-friendly materials (Mathiot et al. 2019; Salehizadeh et al. 2020). Starch is regarded as a readily available renewable organic material served as the feedstock for fermentation-based biofuels (e.g. bioethanol and biobutanol) production and degradable bio-based plastics manufacturing as well as the foodstuff supply (da Maia et al. 2020; Mathiot et al. 2019). Microalgae are considered as ideal alternative starch producers relative to higher plants since they bear many advantages, like high photosynthetic efficiency, flexible and controllable cultivation modes, short growth period, and metabolic plasticity (Aikawa et al. 2015; Chisti 2007). Meanwhile, utilizing algae can avoid competition with terrestrial plants and mitigate CO_2_ in the atmosphere, which could construct a sustainable circular paradigm for biofuels and biorefinery (CO_2_-microalgal starch-biofuels/bio-based chemicals-CO_2_) (Thanigaivel et al. 2022).

Starch accumulates in microalgae normally under stressful conditions, such as nutrition deprivation (usually macroelements, like N, S, and/or P), low salinity, and high irradiance (Brányiková et al. 2011; Ran et al. 2019; Yao et al. 2013b). Nitrogen or sulphur deprivation blocks protein synthesis and DNA replication and thus hinders cells proliferation and directs photosynthetic carbon partitioning in algae from protein to energy reserve substances (e.g. carbohydrate and lipid) (Brányiková et al. 2011). After a period of time suffering from these kinds of stress, intracellular starch can reach 50% dry weight (DW) of algae cells (Yao et al. 2012). However, extreme stress conditions will obstruct the growth of cells that eventually decrease the total biomass and starch yield (Ran et al. 2019; Vitova et al. 2015; Yao et al. 2012). One of the strategies raised to deal with this problem is to adopt a two-stage mode, where in the first stage, the algae cells are cultivated in an optimal condition to achieve high biomass accumulation and then transferred into a stressful condition under a specific physiological status for starch production as the second stage. The initial cell density (ICD) for the second stage is a key factor that affects biomass and starch productivity especially under the photoautotrophic cultivation mode (Carnovale et al. 2021; Cheng et al. 2017; Ivanov et al. 2021; Yao et al. 2013a; Giraldo et al. 2021). High cell density influences the accessibility of light penetration through the culture medium due to the mutual shading, thus altering the mean light intensity that individual algal cell would receive (Brányiková et al. 2011; Carnovale et al. 2021; Yao et al. 2013a). Therefore, a reduced starch content can be usually observed due to the decreased light accessibility, although the biomass and starch yield could be improved (Carnovale et al. 2021; Cheng et al. 2017; Ivanov et al. 2021; Yao et al. 2013a). Moreover, high cell density cultures can often suffer from extra stress, such as photorespiration, because of the oxygen accumulation (Formighieri et al. 2012; Molina et al. 2001) and the excretion of inhibitory metabolites with a prolonged cultivation (Richmond et al. 2003), which could on the contrary impede the biomass and starch production. However, from the industrial point, high cell density is usually necessary in the outdoor cultivation of photoautotrophic microalgae to minimize starch lose at night and maintain starch production ability in the daytime (Brányiková et al. 2011). High cell densities can also improve the economic benefit as it saves the space of photobioreactors which contributes to a considerable part in the production cost (Formighieri et al. 2012). In addition, harvesting (dewatering) and drying costs could also be reduced by applying high cell density cultures (Shen et al. 2009). Therefore, the problem that the reduction of starch content and the possible extra stress that algae could face under high cell density cultures needs to be resolved before the advantages are fully exploited.

The regulation of physiological status in microalgae by the implementation of chemicals, such as phytohormones and antioxidants to relive abiotic stress and improve target substances production, is attracting a growing attention (Ran et al. 2019; Zhao et al. 2019b). γ-Aminobutyric acid (GABA) is a four-carbon non-protein amino acid that has often been suggested as a metabolite or signalling molecule in plants (Seifikalhor et al. 2019). Its biological functions remain mysterious in many aspects, anyhow this compound shows the ability to activate antioxidant defense systems and reduce reactive oxygen species under many abiotic stresses, like salinity, drought, and temperature (Vijayakumari and Puthur 2016). Due to its role in regulating cell growth and enhancing stress resistance in the algae, GBAB was reported to promote biomass, astaxanthin, and lipids production in *Haematococcus pluvialis* exposed to high-light and high-salinity stress, and to enhance biomass and lipid accumulation in *Monoraphidium* sp. QLY-1 under Cd stress and in *Chlorella* (Li et al. 2020, 2021; Xue and Ng 2022; Zhao et al. 2020). Previous work had shown the positive effects of GABA on starch production in a green microalga *Tetraselmis subcordiformis*, but the possible role it played on the photosynthesis and stress regulation seemed ambiguous (Ran et al. 2020).

In these contexts, this research aimed to explore the strategy to economically enhance starch production from CO_2_ in this microalga by increasing initial cell density in combination with photosynthetic physiology and stress regulation by GABA supply. The photosynthetic performance and starch accumulation as well as starch composition under nitrogen deprivation were tracked in both normal ICD (NICD) and high ICD (HICD) conditions with different concentrations of GABA supply. In addition, the net extra benefit from the ICD increase and/or GABA addition for photosynthetic starch production in *T. subcordiformis* was also evaluated.

## Material and methods

### Microalgal strain and culture conditions

*Tetraselmis subcordiformis* FACHB-1751 was isolated from Huanghai Sea, Liaoning Province, China, and maintained by the Freshwater Algae Culture Collection of the Institution of Hydrobiology (FACHB collection), Chinese Academy of Sciences. The strain was cultivated in artificial seawater (ASW) with extra 0.81 g L^−1^ Tris and 0.33 mL L^−1^ glacial acetic acid added as described before (Yao et al. 2012). Algae cells for experiments were collected during the last exponential phase and washed twice with nitrogen-free artificial seawater (ASW-N) to eliminate residual nitrate. After then the cells were inoculated in ASW-N with a NICD of 1.1 g L^−1^ (Ran et al. 2020) and HICD of 2.8 g L^−1^, respectively. GABA was added to both the NICD and HICD cultures with a final concentration of 0, 2.5, 5, and 10 mM, respectively.

The microalgae cells were cultivated photoautotrophically in cylindrical glass bubble photobioreactors (50 mm diameter, 400 mm height) with a working volume of 500 mL under 25 ± 2 ℃ (Yao et al. 2012). A constant 2% CO_2_-rich air was injected into the cultures with a rate of 0.4 vvm. A continuous illumination was provided with cool white fluorescent lamps at an incident light intensity of 100 μmol m^−1^ s^−1^ from one side. All experiments were performed in three biological replicates.

### Growth measurement

The cell growth as revealed by biomass dry weight (DW) was determined gravimetrically according to Yao et al. (2018). The biomass theoretical productivity (*P*_b_, g L^−1^ day^−1^) was calculated as follows:

1 $$P_{{\text{b}}} = \left( {{\text{DW}}_{t} - DW_{0} } \right)/t,$$

where DW_*t*_ and DW_0_ are the biomass dry weight at culture times *t* and 0, respectively.

### Photosynthetic performance analysis

The fast fluorescence induction kinetics (OJIP test) was applied to evaluate the photosynthetic performance of the microalgae with fluorometer Os30p^+^ (Opti-sciences, USA) (Qi et al. 2019). Parameters represent different processes in photosystem II (PSII), namely, the PSII maximum photochemical efficiency (*F*_v_/*F*_m_), relative variable fluorescence at the *J*-step and *I*-step (*V*_*j*_ and *V*_*i*_), the quantum yield of electron transport (ΦEo), and the electron transport flux from *Q*_A_ to *Q*_B_ per reaction centre (ET_0_/RC), were taking into account for estimating the photosynthetic performance. Each parameter was calculated as described by Strasserf and Srivastava (1995):

2 $$F_{{\text{v}}} /F_{{\text{m}}} = \left( {F_{{\text{m}}} {-}F_{0} } \right)/F_{{\text{m}}} ,$$

3 $${\text{ABS}}/{\text{RC}} = M_{0} /\left( {V_{j} \times F_{{\text{v}}} /F_{{\text{m}}} } \right),$$

4 $$V_{j} = \left( {F_{j} {-} \, F_{0} } \right)/\left( {F_{{\text{m}}} {-} \, F_{0} } \right),$$

5 $$V_{i} = \left( {F_{i} {-}F_{0} } \right)/\left( {F_{{\text{m}}} {-}F_{0} } \right),$$

6 $$\Phi {\text{Eo}} = {\text{ET}}_{0} /{\text{ABS}} = \left[ {1{-}\left( {F_{0} /F_{{\text{m}}} } \right)} \right]\left( {1{-}V_{j} } \right),$$

7 $${\text{ET}}_{0} /{\text{RC}} = M_{0} /V_{j} \times \left( {1 - V_{j} } \right),$$

where *F*_v_ represents the variation of chlorophyll fluorescence between maximal fluorescence (*F*_m_) induced by saturating pulse, *F*_0_ represents the initial fluorescence, and *F*_*j*_ and *F*_*i*_ represent the fluorescence at *J* phase and *I* phase, respectively. *M*_0_ represents the approximate value of the initial slope of the relative variable fluorescence. All these parameters were measured after dark adaption for 10 min.

### Starch measurement

The starch accumulated intracellularly was qualitatively visualized via optical microscope (SMZ180-LT, Phoenix, China) after stained with the iodine solution (0.2% *I*_2_, 2% KI) at an algal culture suspension/iodine solution ratio of 1/1 (v/v) as described previously (Yao et al. 2018).

The starch content (%DW) was quantified according to the previous researches with modifications (Qi et al. 2019). In brief, the starch was extracted with 30% perchloric acid through a 30-s stir accompanied with a 5-min interval for three times. After centrifugation, the supernatant was collected and made up to 5 mL with distilled water. The solution was stained with diluted (1:2, v/v) Lugol’s *I*_2_-KI solution at 25 °C for 15 min before measurement spectrophotometrically at 618 nm and 550 nm according to (Hovenkamp-Hermelink et al. 1988). This method allowed for the simultaneous determination of concentrations of amylose (Am) and amylopectin (Ap). The starch quantity equals to the sum of amylose and amylopectin, and the theoretical productivities of starch (*P*_s_, g L^−1^ day^−1^), amylose (*P*_am_, mg L^−1^ day^−1^), and amylopectin (*P*_ap_, mg L^−1^ day^−1^) were estimated as follows:

8 $$P_{{\text{s}}} = \left( {C_{{{\text{st}}}} {-}C_{{{\text{s}}0}} } \right)/t,$$

9 $$P_{{{\text{am}}}} = \left( {C_{{{\text{amt}}}} {-}C_{{{\text{am}}0}} } \right)/t \times 1000,$$

10 $$P_{{{\text{ap}}}} = \left( {C_{{{\text{apt}}}} {-}C_{{{\text{ap}}0}} } \right)/t \times 1000,$$

where the *C*_st_, *C*_amt_, and *C*_apt_ represent the concentration of starch, amylose, and amylopectin at culture time *t*, respectively, and *C*_s0_, *C*_am0_, and *C*_ap0_ represent the concentration of starch, amylose, and amylopectin at culture time 0, respectively.

### Preliminary techno-economic analysis

The benefit from the enhancement of starch production (*B*_s_, $ m^−3^ culture) due to the increase of ICD from NICD to HICD and the addition of GABA was evaluated as follows:

11 $$B_{{\text{s}}} = \left( {P_{{{\text{am}}}} \times {\text{SP}}_{{{\text{am}}}} + \, P_{{{\text{ap}}}} \times {\text{SP}}_{{{\text{ap}}}} } \right) \times \, t/1000,$$

where SP_am_ and SP_ap_ are the selling price ($ kg^−1^) of Am and Ap, respectively. The SP_am_ and SP_ap_ used for calculation are 30 $ kg^−1^ and 20 $ kg^−1^, respectively, obtained from the companies (SP_am_ from Wuhan Lwax Pharma Tech Co. Ltd. and SP_ap_ from Sinoconvoy new material (Shan dong) Co. Ltd. Trading Company).

The net extra benefit from GABA addition and ICD increase (Δ*B*_s_, $ m^−3^ culture) was assessed by eliminating the cost of both GABA and the increased biomass seed, and the value was presented as the relative benefit from starch production compared to that under NICD without GABA addition (NICD-0) as follows:

12 $$\begin{aligned} \Delta B_{{\text{s}}} & = \left[ {B_{{{\text{s}}(n - m)}} - W_{{{\text{GABA}}(n - m)}} \times {\text{SP}}_{{{\text{GABA}}}} - {\text{ICD}}_{(n - m)} \times C_{{{\text{Biomass}}}} } \right] \\ & \quad - \left[ {B_{{{\text{s}}({\text{NICD-}}0)}} - W_{{{\text{GABA}}({\text{NICD-}}0)}} \times {\text{SP}}_{{{\text{GABA}}}} - {\text{ICD}}_{{({\text{NICD-}}0)}} \times C_{{{\text{Biomass}}}} } \right], \\ \end{aligned}$$

where *B*_s(*n*−*m*)_ is the *B*_s_ of the culture under NICD or HICD (expressed as “*n*”) and GABA addition with concentrations of 0, 2.5, 5, or 10 mM (expressed as “*m*”), W_GABA(*n*−*m*)_ (g L^−1^) is the GABA concentration used in the specific “*n*−*m*” culture group, SP_GABA_ ($ kg^−1^) represents the selling price of GABA [11.5 $ kg^−1^ (Xiong et al. 2017)], ICD_(*n*−*m*)_ (g L^−1^) represents the initial cell density used in the specific “*n*−*m*” culture group, and *C*_Biomass_ ($ kg^−1^) represents the production cost of algal biomass.

### Statistical analysis

Results are expressed as mean ± SD from three independent experiments. IBM SPSS Statistics 25.0 software was used to perform the statistical analysis. Multiple group comparisons were performed using one-way analysis of variance (ANOVA) and Fisher’s LSD. Values of *p* < 0.1, 0.05, and 0.01 were defined as weak significance, significance, and strong significance, respectively.

## Results and discussion

### Biomass accumulation

**Fig. 1:**
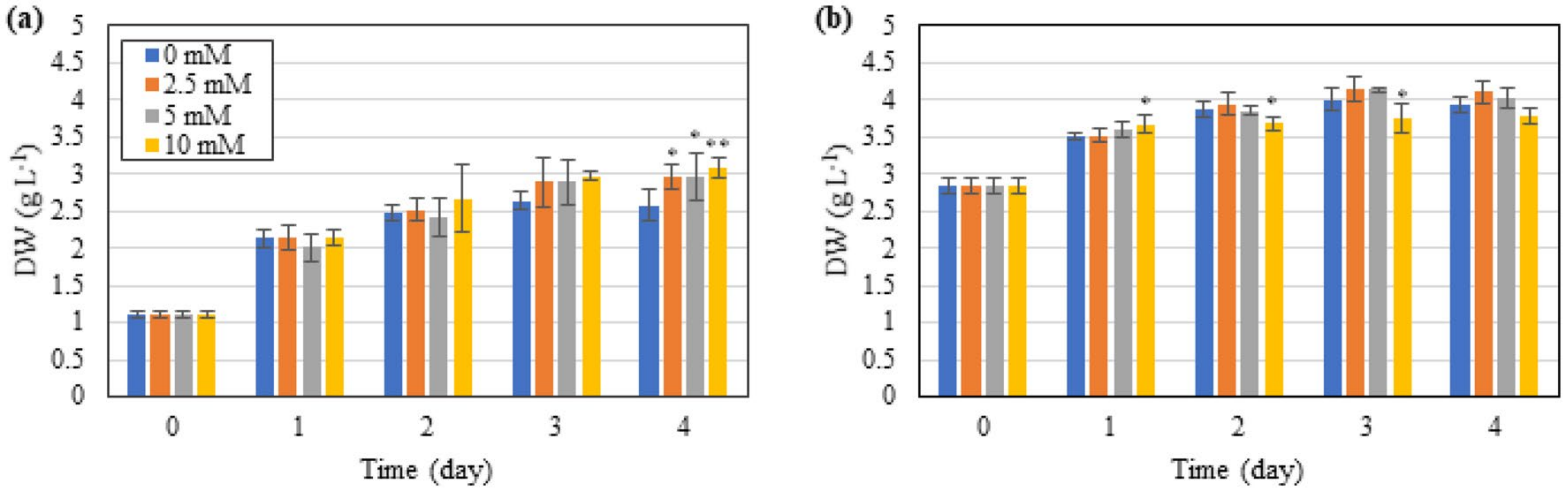
Effect of exogenous GABA supply on biomass accumulation in *T. subcordiformis* exposed to nitrogen deprivation under NICD (**a**) and HICD (**b**). * and ** denote the significant differences at *p* < 0.1 and *p* < 0.05 levels, respectively, under different GABA conditions (2.5–10 mM) compared to those under 0 mM GABA (means ± SD, *n* = 3)

To evaluate the effect of ICD and GABA on the biomass accumulation in *T. subcordiformis* under nitrogen deprivation, the daily dry weight of the cultures was traced. As shown in Fig. 1a, b, although the microalgae were cultivated without extra nitrogen supply, the algal biomass could accumulate continuously for 3 days, which could be ascribed to the recycle of intracellular nitrogen source (such as protein or chlorophylls) to support the short-term CO_2_ fixation for biomass production (Additional file 1: Table S1), as have been demonstrated in early researches in this microalga (Ran et al. 2020; Yao et al. 2012, 2018). Although intracellular nitrate stored in cells could also support the biomass accumulation under nitrogen deprivation, it was speculated to be minor here (contribution of less than 10% of total biomass production, data not shown). The biomass concentration was overall higher under HICD relative to NICD, which coincided with the notion that the final biomass is often positively associated with the initial cell density in microalgae (Dunn and Manoylov 2016). NICD enabled higher biomass theoretical productivity than HICD (Table 1), probably due to the less biomass in the culture and hence more facilitated light penetration under NICD (Chen 1996).

**Table 1:** Biomass, amylose (Am), and amylopectin (Ap) production in *T. subcordiformis* exposed to nitrogen deprivation under NICD and HICD conditions with GABA supply on Day 4

ICD	GABA (mM)	Biomass theoretical productivity (mg L^−1^ day^−1^)	Am/Ap ratio	Am content (%DW)	Am yield (g L^−1^)	Am theoretical productivity (mg L^−1^ day^−1^)	Ap content (%DW)	Ap yield (g L^−1^)	Ap theoretical productivity (mg L^−1^ day^−1^)
NICD	0	366.39 ± 54.20	0.51 ± 0.03	17.81 ± 0.34	0.46 ± 0.05	101.70 ± 11.80	32.79 ± 2.90	0.85 ± 0.13	166.92 ± 32.30
	2.5	462.78 ± 43.41*	0.47 ± 0.03	20.22 ± 1.45	0.60 ± 0.06*	136.79 ± 16.18*	39.38 ± 8.94	1.17 ± 0.28	247.04 ± 69.28
	5	462.22 ± 82.32*	0.42 ± 0.04**	18.52 ± 1.49	0.55 ± 0.11	124.76 ± 26.55	49.86 ± 4.79***	1.49 ± 0.30^***^	326.33 ± 73.99***
	10	494.44 ± 32.50**	0.40 ± 0.01***	16.96 ± 2.61	0.52 ± 0.08	117.86 ± 21.14	51.39 ± 4.78***	1.59 ± 0.16***	351.87 ± 41.03***
HICD	0	274.72 ± 24.07	0.38 ± 0.02	14.84 ± 0.79	0.58 ± 0.04	68.13 ± 11.09	39.39 ± 3.91	1.55 ± 0.19	314.96 ± 46.55
	2.5	316.11 ± 39.06	0.40 ± 0.01**	17.76 ± 3.10*	0.73 ± 0.15*	104.89 ± 37.87*	43.83 ± 7.27	1.80 ± 0.36	378.14 ± 90.08
	5	293.89 ± 33.69	0.43 ± 0.01***	16.84 ± 0.97	0.68 ± 0.06	91.13 ± 15.52	39.26 ± 3.39	1.58 ± 0.19	321.38 ± 47.13
	10	234.17 ± 27.57	0.42 ± 0.01***	14.56 ± 0.91	0.55 ± 0.03	59.27 ± 7.13	34.52 ± 2.02	1.30 ± 0.05	252.20 ± 13.09

*, **, and *** denote the significant differences at *p* < 0.1, *p* < 0.05, and *p* < 0.01 levels, respectively, under different GABA conditions (2.5–10 mM) compared to those under 0 mM GABA (means ± SD, *n* = 3)

Under NICD the biomass accumulation was improved in all the cultures with 2.5–10 mM GABA addition (especially with 10 mM GABA, *p* < 0.05) where a final biomass production of ~ 3.0 g L^−1^ and a theoretical productivity of ~ 0.47 g L^−1^ day^−1^ were achieved on Day 4, which represented 15% and 27% of enhancement, respectively, compared to the one without GABA addition (NICD-0 mM GABA, Fig. 1a, Table 1). However, under HICD, only with low concentration (2.5 mM) of GABA addition could the biomass accumulation be slightly increased, with a maximum biomass production of 4.1 g L^−1^ and theoretical productivity of 0.32 g L^−1^ day^−1^ observed on Day 4, which were 4% and 15% higher, respectively, than the one without GABA addition (HICD-0 mM GABA, Fig. 1b, Table 1). The biomass accumulation was even decreased by 15% under GABA addition with a high concentration (10 mM) relative to the 0 mM GABA counterpart, indicating that high concentration of GABA exerted inhibition on algae biomass production (Fig. 1b, Table 1). In general, the positive regulation of biomass production by GABA addition was more pronounced under NICD rather than HICD. However, our previous study conducted with NICD under a relatively higher light intensity (150 vs. 100 μmol m^−2^ s^−1^) revealed a marginal effect of GABA addition on biomass accumulation under nitrogen deprivation, but showed significant inhibition under nitrogen limitation (Ran et al. 2020). The discrepancy between the two ICD conditions herein along with the distinct effects found in our previous study suggested the complexity of GABA regulation on microalgae physiology under different environmental conditions. Nevertheless, proper GABA supply could facilitate biomass production in *T. subcordiformis*, especially under NICD.

### Photosynthetic performance

Biomass accumulation and starch production are closely related to photosynthesis in photoautotrophic microalgae and are sensitive to stress conditions as well (Ran et al. 2019). The fast chlorophyll a fluorescence transient (OJIP) is one of the effective methods for characterizing the photosynthetic energy conversion in photosystem II, which has been widely used for monitoring responses to stressors in plants and algae (Stirbet 2011). Here the OJIP test was applied to dissect the photosynthetic performance of the microalgae exposed to nitrogen deprivation under different ICDs and to show the potential regulatory effect of GABA on the stress and photosynthesis.

#### Overall photosynthetic activity

**Fig. 2:**
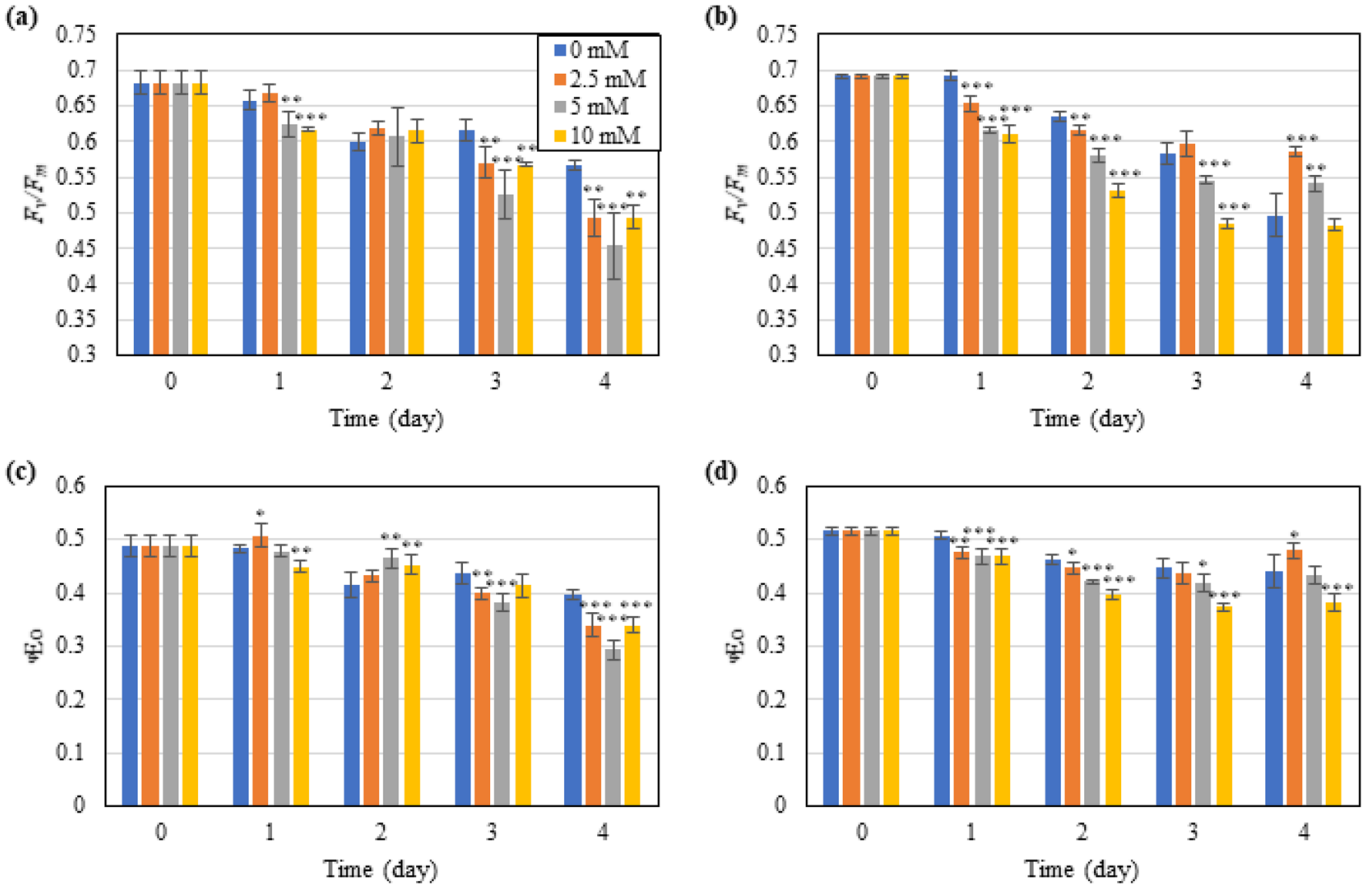
Effect of exogenous GABA supply on the maximum photochemical efficiency (*F*_v_/*F*_m_, **a**, **b**) and quantum yield of electron transport (ΦEo, **c**, **d**) in *T. subcordiformis* exposed to nitrogen deprivation under NICD (**a**, **c**) and HICD (**b**, **d**). *, **, and *** denote the significant differences at *p* < 0.1, *p* < 0.05, and *p* < 0.01 levels, respectively, under different GABA conditions (2.5–10 mM) compared to those under 0 mM GABA (means ± SD, *n* = 3)

The overall photosynthetic performance can be evaluated as quantum yield of the photosystems. In general, *F*_v_/*F*_m_ represents the maximum quantum yield of photosystem II (PS II) and is often used as a stress indicator (Stirbet 2011). As the stress intensifying, *F*_v_/*F*_m_ will be gradually declining in most cases which indicates the reduction of photosynthesis by algae cells (Zhao et al. 2017). As demonstrated in Fig. 2a, b, *F*_v_/*F*_m_ showed an overall decline under both ICDs exposed to nitrogen deprivation. Under the nitrogen-depleted condition, the photosynthetic activity mostly declined because the synthesis of proteins is impeded (as demonstrated by the decreased protein content, Additional file 1: Table S1), and the photosynthesis-essential proteins, like D1 protein and ribulose bisphosphate carboxylase/oxygenase, will be recycled for nitrogen turnover (Park et al. 2015). The *F*_v_/*F*_m_ level declined sharper in HICD (from 0.69 to 0.50) than in NICD (from 0.68 to 0.57) within 4 days in the cultures without GABA addition (0 mM GABA). Normally, high cell density is supposed to cause less light exposure per algae cell which conducts to less damage to photosynthesis system (Yao et al. 2018). However, it did not apply in the present study. There might be some other factors, such as the increased photorespiration (Formighieri et al. 2012; Molina et al. 2001) or excreted inhibitory metabolites (Richmond et al. 2003) under HICD, which aggravated the stress. Interestingly, GABA played different roles under different ICD conditions, as shown by *F*_v_/*F*_m_. Under NICD, GABA addition with all the concentrations tested significantly (*p* < 0.05) accelerated the decline of *F*_v_/*F*_m_, with ~ 80% of the level remained compared to that without GABA addition (0 mM GABA), which manifested an enhancement of stress (Fig. 2c). Under HICD, GABA showed a similar effect as under NICD in the first 2 days, but it retarded the decrease of *F*_v_/*F*_m_ from Day 3 to Day 4, especially with low GABA dosage (2.5 mM GABA), indicating that GABA with this concentration alleviated the stress. Notably, the protective effect of GABA on *F*_v_/*F*_m_ receded beyond 2.5 mM with a dosage-dependent manner, indicating that high concentration of GABA was inhibitory to photosynthesis.

ΦEo refers to the quantum yield of electron transport between two photosystems (PSII and PSI), which generally decreases when cells are exposed to stressful conditions (Xiang et al. 2021; Zhao et al. 2017). As shown in Fig. 2c, d, for *T. subcordiformis* here, both in NICD and HICD, the variation of ΦEo almost mirrored that of *F*_v_/*F*_m_, i.e. GABA addition under NICD reduced the ΦEo by 14–26%, while conversely under HICD 2.5 mM GABA addition increased the level compared to the cultures with no GABA addition on Day 4, indicating the inhibitory and stimulatory effects of GABA on photosynthetic efficiency under NICD and HICD conditions, respectively. These results differed from the general acknowledgement that GABA usually entitles stress tolerance in microalgae and plants in response to unfavourable conditions, such as high light, high salt, and heavy metal exposure (Li et al. 2020, 2021; Zhao et al. 2020). Here in the alga *T. subcordiformis* exposed to nitrogen deprivation, it highlighted the possibility of different roles that GABA could play on the photosynthetic physiology under different ICD conditions.

#### Light harvesting and photosynthetic electron transport

**Fig. 3:**
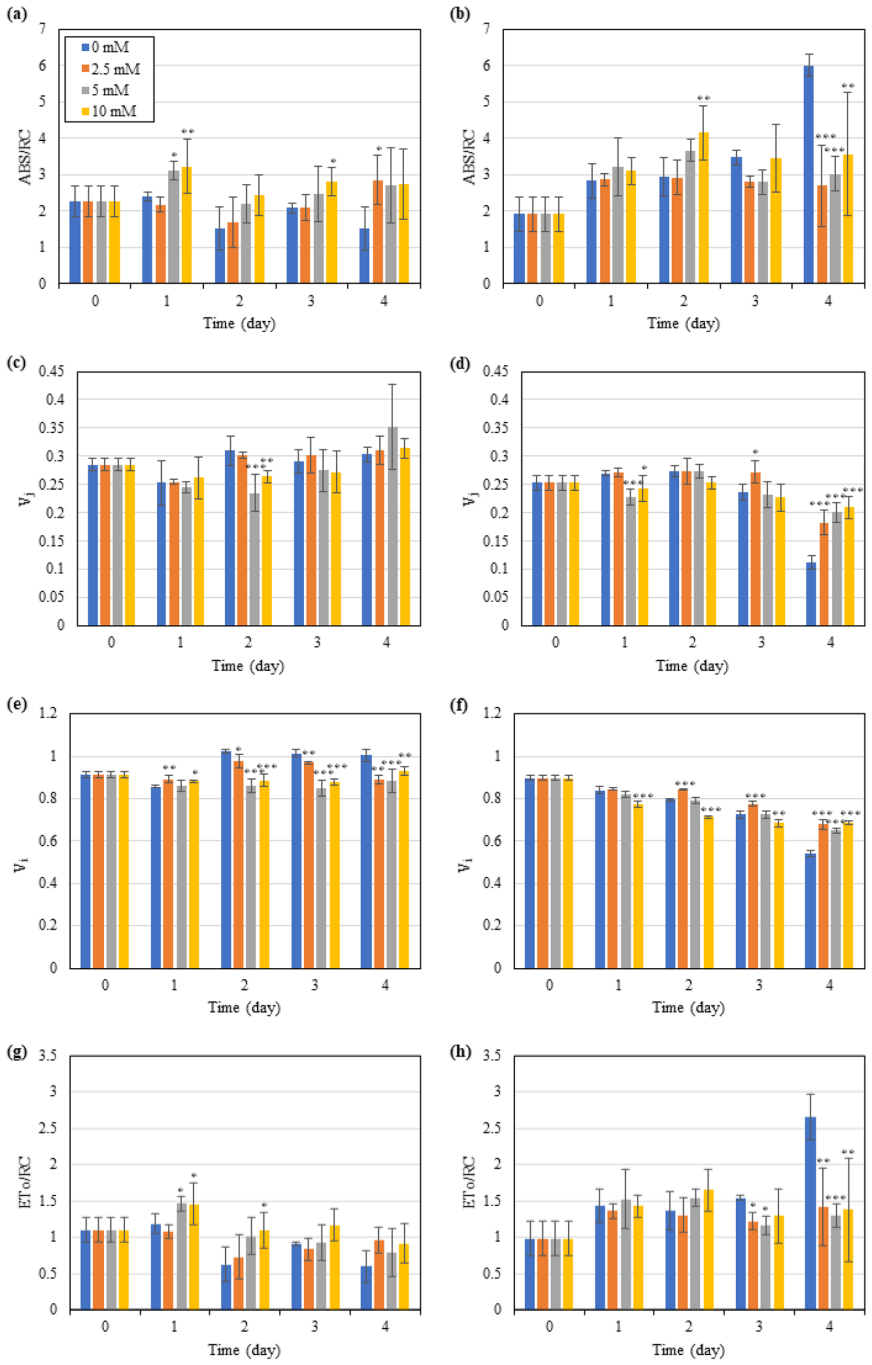
Effect of exogenous GABA supply on the specific energy fluxes per fully active PSII reaction centre (ABS/RC, **a**, **b**), relative variable fluorescence at the *J*-step (*V*_*j*_, **c**, **d**) and *I*-step (*V*_*i*_, **e**, **f**), and electron transport flux per RC (ET_0_/RC, **g**, **h**) in *T. subcordiformis* exposed to nitrogen deprivation under NICD (**a**, **c**, **e**, and **g**) and HICD (**b**, **d**, **f**, and **h**). *, **, and *** denote the significant differences at *p* < 0.1, *p* < 0.05, and *p* < 0.01 levels, respectively, under different GABA conditions (2.5–10 mM) compared to those under 0 mM GABA (means ± SD, *n* = 3)

To further dissect the functional changes of photosynthetic apparatus in *T. subcordiformis* under different ICDs and GABA exposure, the light harvesting and photosynthetic electron transport were measured in detail. The specific energy fluxes per fully active PSII reaction centre (ABS/RC) represents the antenna size of the reaction centre, which reflects the light harvesting ability. It often increases under stressful conditions, such as extreme CO_2_, nitrogen starvation, high nitrate, and high ammonia, due to the inactivation of PSII centres which transfer their antenna to the remaining active PSII (Papazi et al. 2008; Xiang et al. 2021; Zhao et al. 2017, 2019a). Under NICD without GABA addition, ABS/RC showed marginal variations, suggesting an unchanged light harvesting ability and a relatively minor stress the algae were subjected to (Fig. 3a). GABA addition increased the ABS/RC (Fig. 3a) under NICD, indicating that it entitled more excessive energy absorbed by PSII that could result in aggravated photodamage under nitrogen deprivation, as demonstrated by declined *F*_v_/*F*_m_ and ΦEo (Fig. 2a, c). As for HICD, ABS/RC showed an overall increase in the culture without GABA addition under nitrogen deprivation (especially on Day 4, Fig. 3b), which differed from the performance under NICD where it remained stable (Fig. 3a). It suggested that the algae under HICD were subjected to a severer stress than that under NICD, which was in accordance with the lower *F*_v_/*F*_m_ (Fig. 2a, b). During the first 2 days when the algae were still under a moderate stress in the HICD culture (*F*_v_/*F*_m_ > 0.6), GABA acted similar to that under NICD that increased the ABS/RC and hence strengthened the stress; however, GABA addition significantly reduced (*p* < 0.01 for 2.5 and 5 mM GABA, *p* < 0.05 for 10 mM GABA) the ABS/RC on Day 4, indicating that GABA could protect the algal cells in turn by decreasing antenna size to reduce photodamage when severe stress occurred (Fig. 3b).

**Fig. 4:**
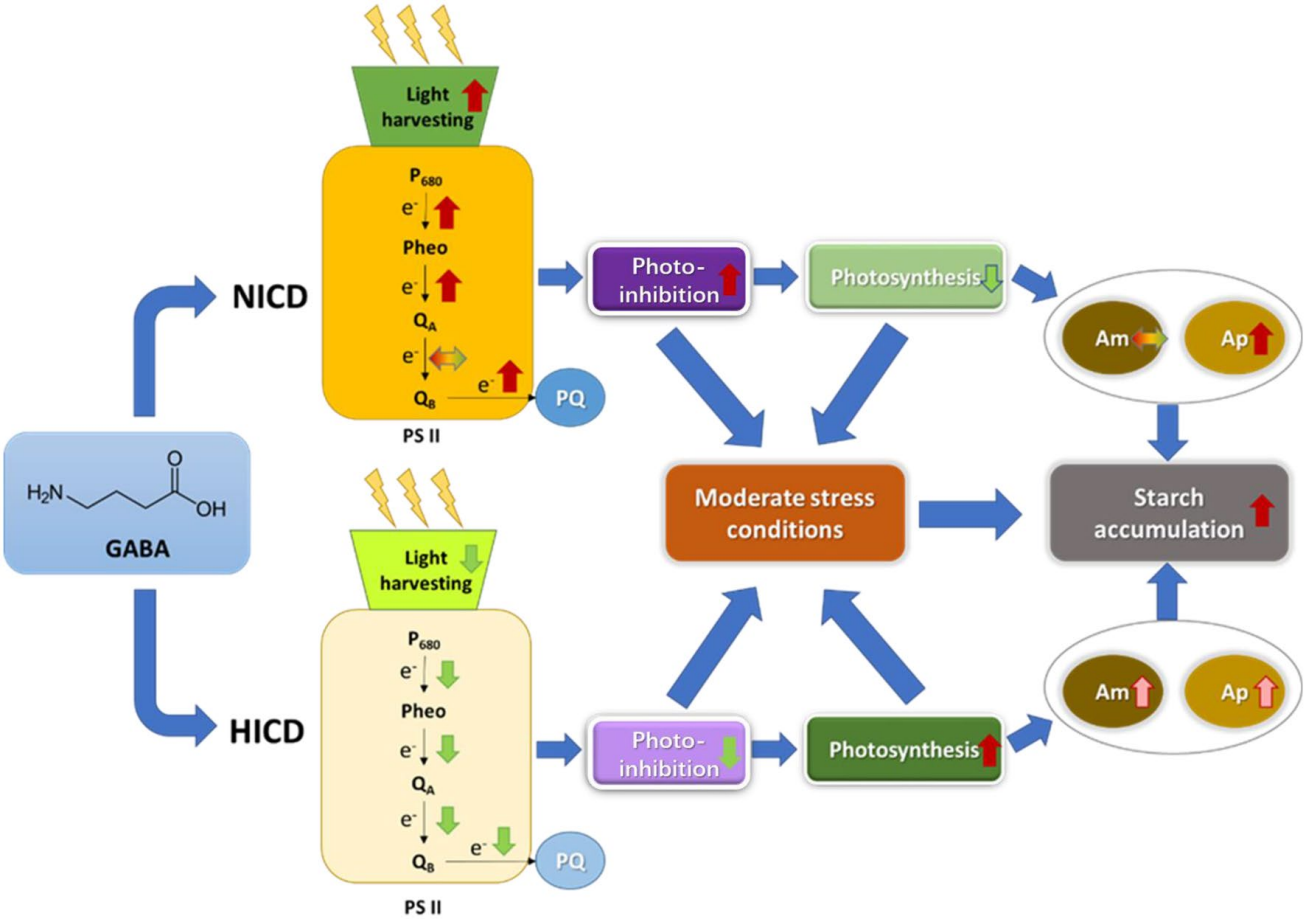
Summary of the different effects of GABA on the photosynthetic physiology, stress regulation, and starch accumulation in *T. subcordiformis* exposed to nitrogen deprivation under NICD and HICD conditions. The summary was based on the results obtained on Day 4 under NICD with 0 vs. 5 mM GABA and HICD with 0 vs. 2.5 mM GABA which showed maximum starch production improvements, respectively

For the evaluation of downstream photosynthetic electron transport status in PS II, two parameters, *V*_*j*_ and *V*_*i*_, were introduced. *V*_*j*_, which represents the relative variable fluorescence at the *J*-step, reflects the reduction status of the primary electron acceptor *Q*_A_ in a form of *Q*_A_^−^*Q*_B_; *V*_*i*_ is the relative variable fluorescence at *I*-step, which reflects the reduction process of both *Q*_A_ and *Q*_B_ to *Q*_A_*Q*_B_^2−^ or *Q*_A_^−^*Q*_B_^2−^ (Malapascua et al. 2014; Strasserf and Srivastava 1995). The increase of *V*_*j*_ and *V*_*i*_ is regarded as the blockage of the electron transfer from *Q*_A_ to *Q*_B_ and from *Q*_B_ to the downstream acceptor PQ pool, respectively, and vice versa (Park et al. 2015; Zhao et al. 2017). They were often found to increase when microalgae were exposed to stress conditions, like nitrogen deprivation, suboptimal temperature, and toxic chemicals (Benavides et al. 2017; Kamalanathan et al. 2016; Xiang et al. 2018). As shown in Fig. 3c, e, for *T. subcordiformis* under NICD without GABA addition, *V*_*i*_ showed an overall slight increase under nitrogen deprivation, which corresponded to the general stress response described above, while *V*_*j*_ remained almost constant. However, under HICD, *V*_*i*_ decreased continuously when exposed to nitrogen deprivation, whereas *V*_*j*_ level fell on Days 3 and 4 (Fig. 3d, f), which indicated a different response mode compared to that under NICD. Moreover, GABA addition also showed distinct effects on *V*_*j*_ or *V*_*i*_ under NICD and HICD. As shown in Fig. 3c–f, under NICD, GABA addition mainly mitigated the increase of *V*_*i*_ (Day 4, *p* < 0.05), while it attenuated the decline of both *V*_*j*_ and *V*_*i*_ under HICD (Day 4, *p* < 0.01). These results suggested that GABA accelerated the electron transport from *Q*_B_ to PQ pool under NICD, but conversely it impeded the entire electron transport from *Q*_A_ to PQ pool in PSII under HICD. In addition, another parameter ETo/RC representing electron transport flux per active reaction centre showed similar profile as ABS/RC and reverse action as *V*_*i*_ (Fig. 3g, h), further demonstrating that GABA acted as a stimulator on electron transfer in PSII under NICD, but as an inhibitor under HICD. It should be noted that the facilitation of photosynthetic electron transfer under NICD by GABA seemed to be corresponded to the aggravation of stress (declined *F*_v_/*F*_m_ and ΦEo, and increased ABS/RC, as discussed above) instead of alleviation, and vice versa under HICD. In fact, the photosynthetic electron-transport chains (ETC) in PSII, especially from *Q*_A_^−^*Q*_B_^−^PQ, tends to produce reactive oxygen species (ROS) that is damaging to photosynthetic apparatus and causes photoinhibition in chloroplast (Edreva 2005). Taken together, it suggested that *T. subcordiformis* cultivated with HICD under nitrogen deprivation suffered from severer stress than that with NCID; GABA tended to increase the antenna size and accelerated the electron transfer in the reaction centre of PSII and also from it to the downstream acceptor PQ pool, which resulted in excessive energy absorption and processing that led to strengthened photoinhibition under NICD, while it protected photosynthesis in a reverse way under HICD (Fig. 4).

### Starch accumulation

#### Overall starch production

**Fig. 5:**
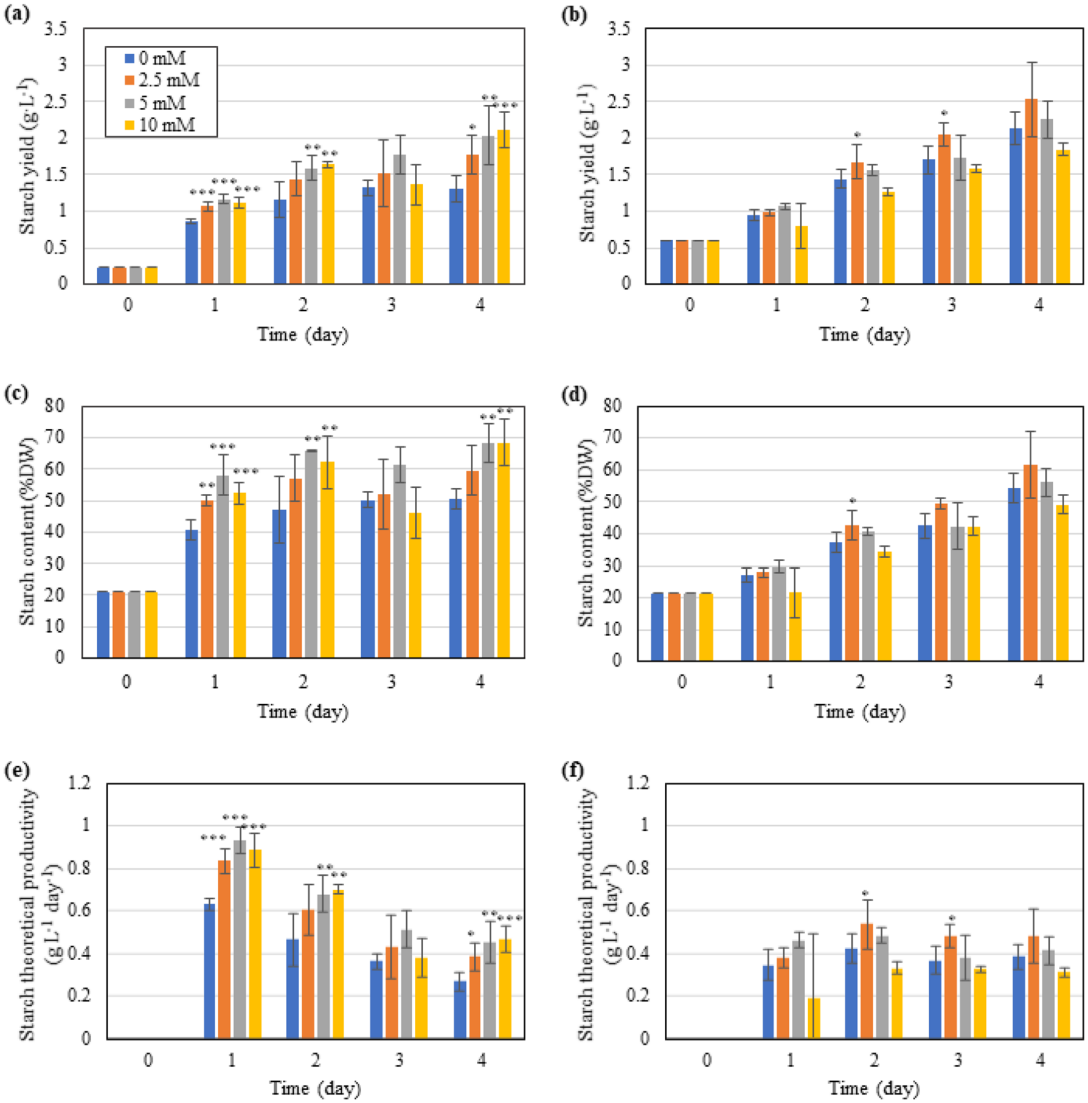
Effect of exogenous GABA supply on the starch content (**a**, **b**), yield (**c**, **d**), and theoretical productivity (**e**, **f**) in *T. subcordiformis* exposed to nitrogen deprivation under NICD (**a**, **c** and **e**) and HICD (**b**, **d** and **f**). *, **, and *** denote the significant differences at *p* < 0.1, *p* < 0.05, and *p* < 0.01 levels, respectively, under different GABA conditions (2.5–10 mM) compared to those under 0 mM GABA (means ± SD, *n* = 3)

**Fig. 6:**
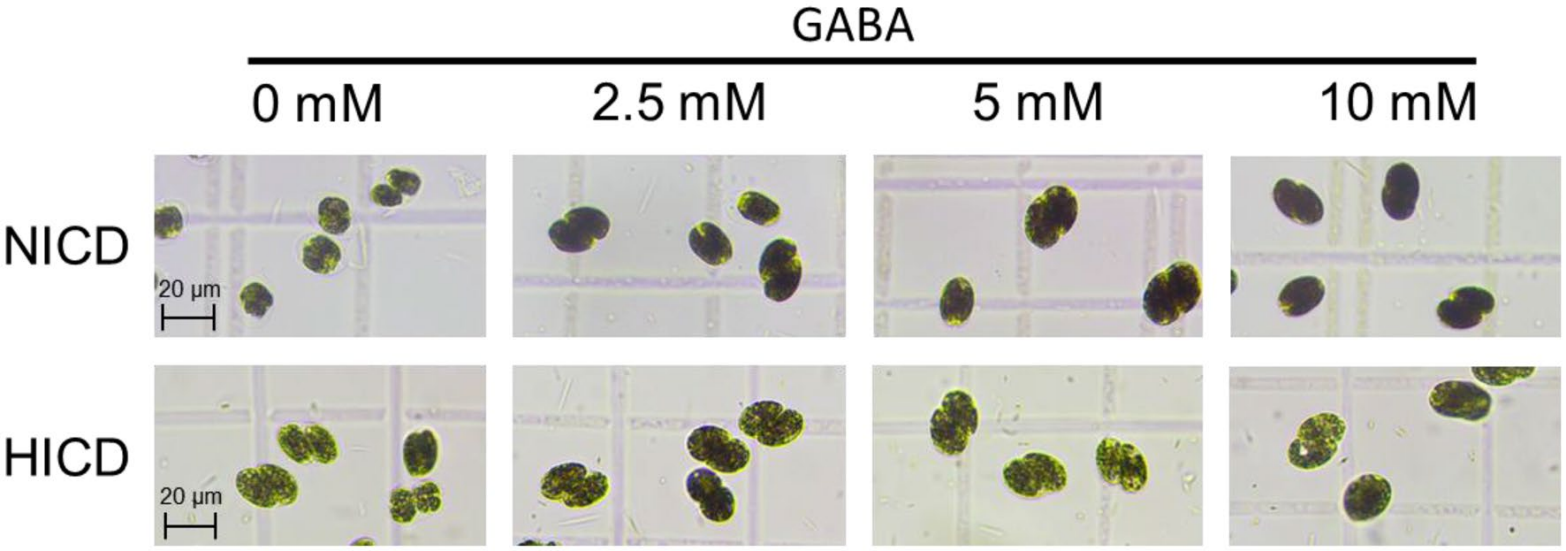
Starch accumulation as revealed by iodine staining in *T. subcordiformis* exposed to nitrogen deprivation under NICD and HICD conditions with GABA supply on Day 4. *, **, and *** denote the significant differences at *p* < 0.1, *p* < 0.05, and *p* < 0.01 levels, respectively, under different GABA conditions (2.5–10 mM) compared to those under 0 mM GABA (means ± SD, *n* = 3)

Nitrogen deprivation can induce starch accumulation in *T. subcordiformis* (Ran et al. 2020), as was also demonstrated here shown in Figs. 5 and 6. Different ICDs could influence starch accumulation, and the effect of GABA on starch production varied under different ICDs as well. In general, NICD led to faster starch accumulation than HICD in the early phase of nitrogen deprivation (0–2 days), with starch content reaching 47.0% and 37.4% under NICD and HICD on Day 2 in the cultures without GABA addition, respectively (Fig. 5a, b). It is acknowledged that lower microalgae cell density could result in higher irradiance accessibility, which exerts more intensive stress under nutrient deprivation conditions and is thus beneficial to starch accumulation (Chen et al. 2015; Yao et al. 2013a). In fact, severer photoinhibition as represented by a faster decline of *F*_v_/*F*_m_ and ΦEo (Fig. 2a–d) could be detected under NICD relative to HICD, which was in aligned with the better starch accumulation under NICD. However, with the cultivation time prolonged, the starch accumulation under HICD exceeded NICD because of the improved stress in the later phase of cultivation (as discussed in “Photosynthetic performance” section). Without GABA addition, the final starch content, yield, and theoretical productivity under HICD reached 54.2%DW, 2.1 g L^−1^, and 0.38 g L^−1^ day^−1^, respectively, on Day 4, which were 7%, 63%, and 42% higher, respectively, than those under NICD (Fig. 5).

GABA addition with concentrations of 2.5–10 mM under NICD facilitated starch production, as demonstrated by the enhanced starch content, yield, and theoretical productivity (Fig. 5a, c, e). Iodine staining of the algae cells also confirmed the improvement of starch accumulation as presented by darker cells (Fig. 6). The maximum starch content of 68.4% and starch yield of 2.1 g L^−1^ were both achieved with medium or high (5 mM or 10 mM) GABA addition on Day 4, which were 35% and 62% higher than those without GABA, respectively (Fig. 5a, c). Similarly, under HICD, although the effects were not as significant as that under NICD, GABA addition with low concentration still showed an overall improvement in starch production (Figs. 5b, d, 6), with the highest starch content of 61.6% and starch yield of 2.5 g L^−1^ obtained with 2.5 mM GABA addition on Day 4, which represented 14% and 19% of enhancements, respectively, compared with the culture without GABA treatment. However, medium or high concentration of GABA (5–10 mM) seemed unfavourable for starch accumulation under HICD (Fig. 5b, d, f), probably due to their inhibitory effects on photosynthesis, as exhibited by declined biomass production and *F*_v_/*F*_m_ with a dose-dependent manner (Figs. 1b and 2b).

It is believed that stress is indispensable for starch accumulation in microalgae as it causes carbon flux directed into carbohydrate instead of protein synthesis under unfavourable conditions, but adequate photosynthesis is a prerequisite for sustained starch production as well (Ran et al. 2019, 2020; Yao et al. 2012). Here, although proper GABA addition resulted in enhanced starch production in both NICD (2.5–10 mM GABA) and HICD (2.5 mM GABA) conditions, the underlying reasons seemed distinct. The enhanced starch production by GABA addition under NICD could be ascribed to the intensified stress, as demonstrated by lower *F*_v_/*F*_m_ and ΦEo with GABA addition described in Fig. 2a, c. Conversely, under HICD, considering that higher cell density resulted in stronger stress than NICD (lower *F*_v_/*F*_m_ on Day 4, probably due to the extra stress effect from photorespiration or excreted inhibitory metabolites, as discussed in “Overall photosynthetic activity” section), it could be the protective effect of GABA against photoinhibition that maintained sufficient photosynthetic activity (higher *F*_v_/*F*_m_ and ΦEo, 2.5 mM GABA compared with 0 mM GABA, Fig. 2b, d) and consequently improved starch accumulation. It should be mentioned that the stress intensification effect of GABA occurred under NICD and also the first 2 days of HICD when minor stress was present (*F*_v_/*F*_m_ higher than 0.6, Fig. 2a, b), whereas the protective effect of GABA (2.5 mM) appeared in the phase that microalgae were subjected to severe stress (HICD, Day 4, *F*_v_/*F*_m_ lower than 0.5, Fig. 2b). In addition, the previous study demonstrated that GABA tended to exert extra stress in *T. subcordiformis* under normal nutrient conditions (Ran et al. 2020). Collectively, these results exemplified the dual function of GABA on microalgae, the dominance of which could be stress status dependent: GABA could strengthen the stress when it is minor while alleviate it when the stress is in excess. Consequently, GABA could regulate the stress status of microalgae to enable a suitable photosynthetic activity that facilitates starch production (Fig. 4).

#### Starch composition

Starch is generally classified into two categories: amylose (Am) and amylopectin (Ap), the former being linear and the latter being more branched (Aikawa et al. 2015). The proportion of these two components in starch determines the physiochemical properties which is of importance for the potential applications (Altayan et al. 2021). Different ICDs and GABA addition could not only impact starch productivity but also have influence on the relative starch composition. As shown in Table 1, without GABA addition (0 mM), the Am content and theoretical productivity under HICD decreased by 17% and 33%, respectively, while the Ap content and theoretical productivity increased by 20% and 89%, respectively, compared to those under NICD, leading to 25% of decline in Am/Ap ratio (0.38 in HICD vs 0.51 in NICD). It indicated that Ap production was more favoured under HICD relative to NICD. However, GABA addition reversed this profile. As shown in Table 1, GABA addition significantly reduced (*p* < 0.05 for 5 mM GABA, *p* < 0.01 for 10 mM GABA) the Am/Ap ratio from 0.51 to 0.40 with a dose-dependent manner from 0 to 10 mM GABA under NICD, while it showed a slight but significant (*p* < 0.05 for 2.5 mM GABA, *p* < 0.01 for 5 mM and 10 mM GABA) increase under HICD (Table 1). It was notably that under NICD, 5 mM and 10 mM GABA addition significantly enhanced (*p* < 0.01) the Ap content by approximately 52–57% compared with that without GABA addition, while Am content remained almost constant (Table 1), indicating that it was the enhancement of Ap rather than Am accumulation that finally led to the improved overall starch production as well as increased relative Ap proportion with GABA addition under NICD (Fig. 5a, c). In contrast, under HICD, Am content was increased when 2.5 mM GABA was added into the culture (Table 1), which accounted for the improvement of starch production (Fig. 5b, d) and Am/Ap ratio. Here, it demonstrated again that GABA exhibited distinct effects on the regulation of starch composition under different ICD conditions. The possible reason could be the different regulatory function of GABA on the stress status therein. In fact, a negative correlation (*R*^2^ = 0.37–0.41) between *F*_v_/*F*_m_ and relative Ap production (Ap theoretical productivity/total starch theoretical productivity) could be found under both NICD and HICD conditions (Additional file 1: Fig. S1), suggesting that severer stress mainly facilitated Ap accumulation. It was also reported that weak-light stress and drought stress could cause an increase of Ap ratio in maize and wheat starch, respectively (Shi et al. 2018; Yu et al. 2016), which was consistent with the results in *T. subcordiformis* herein. Stress could trigger the variations of the activity of key enzymes involved in starch biosynthesis, leading to the alteration of Am/Ap ratio (Lu et al. 2019). As discussed previously, GABA addition strengthened the stress under NICD and vice versa under HICD. Therefore, the regulatory effects of GABA on the relative starch composition should be reasonably different.

Overall, by applying HICD with 2.5 mM GABA supply, the Ap production was highly boosted, with 34%, 112%, and 127% increase of Ap content, yield, and theoretical productivity, respectively, compared with those under NICD without GABA addition (Table 1). Although the Am production was also improved, the relative composition as represented by Am/Ap ratio was finally decreased to 0.38 (Table 1). This Am/Ap ratio was similar to the starch in the native cereal crops, and especially resembled the corn starch (0.39) which is favourable for fermentation to produce liquid biofuels (such as bioethanol) and biomaterials (such as bionanocomposite films for food packaging) (Jha 2021; Tanadul et al. 2014). The efficiently produced Ap (regarded as waxy starch) could be a sustainable supplementary or even substitute for crop-origin waxy starch that can be used in food technologies (e.g. confectionary or bakery) to minimize retrogradation, and also for non-food applications, such as fillers and reinforcing agents in polymer composites, carriers for drug delivery, barrier coating materials, and stabilizers in oil-in-water emulsions (Šárka and Dvořáček 2017). The enhanced Am content and theoretical productivity (Table 1) under HICD with 2.5 mM GABA supply could also contribute to the application of microalgal starch in the field of health products, such as resistant starch, which is composed of high amylose with a function of reducing the glycaemia level in the human body (Birt et al. 2013).

The present study demonstrated that in *T. subcordiformis* exposed to nitrogen deprivation, increasing ICD up to 2.8 g L^−1^ could significantly enhance final starch yield (2.1 vs. 1.3 g L^−1^) and starch theoretical productivity (0.38 vs. 0.27 g L^−1^ day^−1^) without affecting starch content (or even slightly increased from 51 to 54%DW) compared to that with a lower ICD of 1.1 g L^−1^ (Fig. 2 and Table 2, 0 mM GABA). This relationship between ICD and starch production herein was different from the case in *T. subcordiformis* under phosphorus deprivation that increasing ICD from 1.0 to 3.0 g L^−1^ led to decreased starch yield, productivity, and content (Yao et al. 2013a) (Table 2). It was also uncommon among other microalgae (e.g. *Chlorella vulgaris* (Brányiková et al. 2011; Carnovale et al. 2021)) under nutrient deprivation conditions where increasing ICD would normally decrease the starch content (Table 2) due to the reduced mean light intensity. In *Chlorella* sp. AE10, the highest carbohydrate content was achieved under the lowest ICD applied with unaffected carbohydrate concentration and productivity, and hence, low ICD (0.1 g L^−1^) was recommended to induce starch accumulation under nitrogen starvation in that case (Cheng et al. 2017). In contrast, the present study highlighted a HICD condition that could be more favourable for *T. subcordiformis* to produce starch under nitrogen deprivation. In addition, 2.5 mM GABA addition under HICD resulted in 25% higher starch yield and 7% higher theoretical productivity compared to those under NICD with 5 mM GABA addition in which best starch production was achieved therein (Table 2), suggesting that GABA induction for starch accumulation was more efficient under HICD with even less GABA supplied than that under NICD. In fact, it could be calculated that the net increase of starch yield and starch theoretical productivity per millimole of GABA used under HICD was 159.91 mg mmol^−1^ and 39.98 mg day^−1^ mmol^−1^, respectively, which were both higher than those under NICD with 5 or 10 mM GABA addition tested herein (Table 3).

**Table 2:** Comparison of starch production in different microalgae under batch culture mode with different ICDs and nutrient deprivation conditions

Strain	ICD (g L^−1^)	Culture setup and conditions	Starch inducer	Starch content (%DW)	Starch yield (g L^−1^)	Starch theoretical productivity (g L^−1^ day^−1^)	References
*Chlorella* sp. AE10	0.1	350 mL tube PBR, 10% CO_2_, 1000 μmol m^−1^ s^−1^ continuous irradiance	–N (4.4 mM)	60.5	1.21	0.73	Cheng et al. (2017)
*Chlorella* sp. AE10	0.1	As above	–N (4.4 mM)	56.9	1.42	0.71	Yuan et al. (2018)
*Chlorella vulgaris* Beijerinck CCALA924	0.1	300 mL glass cylinder PBR, 2% CO_2_, 780 μmol m^−1^ s^−1^ continuous irradiance	–N	37	0.10	0.19	Brányiková et al. (2011)
	0.1		–P	53	0.35	0.48	
	0.1		–S	60	0.62	0.74	
	0.5		–S	60	1.2	2.32	
	1.2		–S	55	1.8	3.41	
	1.8		–S	40	1.4	2.51	
*Chlorella vulgaris* SAG 211-11b	0.25	1.5 L flat panel PBR, 5% CO_2_, 1300 μmol m^−1^ s^−1^ continuous irradiance	–N	28.9	0.17	0.25	Carnovale et al. (2021)
	0.65		–N	15.0	0.17	0.42	
*Chlorella fusca*	1	2 L flat Roux bottle, 1.5% CO_2_, 120 μmol m^−1^ s^−1^ continuous irradiance	–N	49	1.86	0.38	Jerez et al. (2016)
	1		–S	45	2.03	0.47	
*Tetraselmis subcordiformis*	0.5	500 mL glass column PBR, 2–3% CO_2_, 100–200 μmol m^−1^ s^−1^ continuous irradiance	–N	54.0	0.7	0.49	Yao et al. (2012)
	0.5		–S	62.1	1.2	0.62	
	0.5		–P	44.1	1.1	0.21	Yao et al. (2013a)
	1.0		–P	42.2	1.6	0.30	
	2.0		–P	28.7	1.3	0.19	
	3.0		–P	24.8	1.4	0.18	
	0.5		–N + 3 mM P	64.5	1.0	0.5	Yao et al. (2018)
	0.6		–N + 12 mM NaHCO_3_	58.1	1.5	0.73	Qi et al. (2019)
	1.1		–N	50.6	1.3	0.27	This study
	1.1		–N + 5 mM GABA	68.4	2.0	0.45	This study
	2.8		–N	54.2	2.1	0.38	This study
	2.8		–N + 2.5 mM GABA	61.6	2.5	0.48	This study

**Table 3:** Net increase (Δ) of starch yield and starch theoretical productivity based on GABA supply and net extra benefit from GABA addition and ICD increase in *T. subcordiformis* exposed to nitrogen deprivation under NICD and HICD conditions on Day 4

ICD	GABA (mM)	ΔStarch yield/GABA (mg mmol^−1^)	ΔStarch theoretical productivity/GABA (mg day^−1^ mmol^−1^)	Benefit from starch production ($ m^−3^ culture)	Net extra benefit from GABA addition and ICD increase^a^ ($ m^−3^ culture)
NICD	0	–	–	25.56	0
	2.5	184.34	46.08	36.18	7.65
	5	145.98	36.50	41.08	9.59
	10	80.44	20.11	42.29	4.87
HICD	0	–	–	33.37	3.14
	2.5	159.91	39.98	42.84	9.64
	5	23.53	5.88	36.65	0.48
	10	− 28.65	− 7.16	27.29	− 5.91

^a^The net extra benefit was estimated by assuming a biomass seed production cost of 2.71 $ kg^−1^ (Muhammad et al. 2021) and GABA cost of 11.5 $ kg^−1^ (Xiong et al. 2017)

### Preliminary techno-economic analysis

Further considering the economy of the strategy proposed in the present study that increasing ICD and GABA addition for enhanced starch production, a brief techno-economic analysis was performed based on the laboratory-scale starch production data. Since starch composition (Am and Ap) could influence the selling price of the starch (Zhao et al. 1998), the calculation also incorporated the Am and Ap theoretical productivity separately as a contribution (“Preliminary techno-economic analysis” section). It was shown that the benefit from starch production was enhanced from 25.56 to 33.37 $ m^−3^ culture by singly increasing ICD from 1.1 to 2.8 g L^−1^ (Table 3, 0 mM GABA). Addition of GABA could increase the benefit under both NICD and HICD, with the highest benefit (42.84 $ m^−3^ culture) obtained under HICD with 2.5 mM GABA supply. These results were expected since the benefit was mainly dependent on the starch theoretical productivity, although the Am/Ap could make an influence as well.

**Fig. 7:**
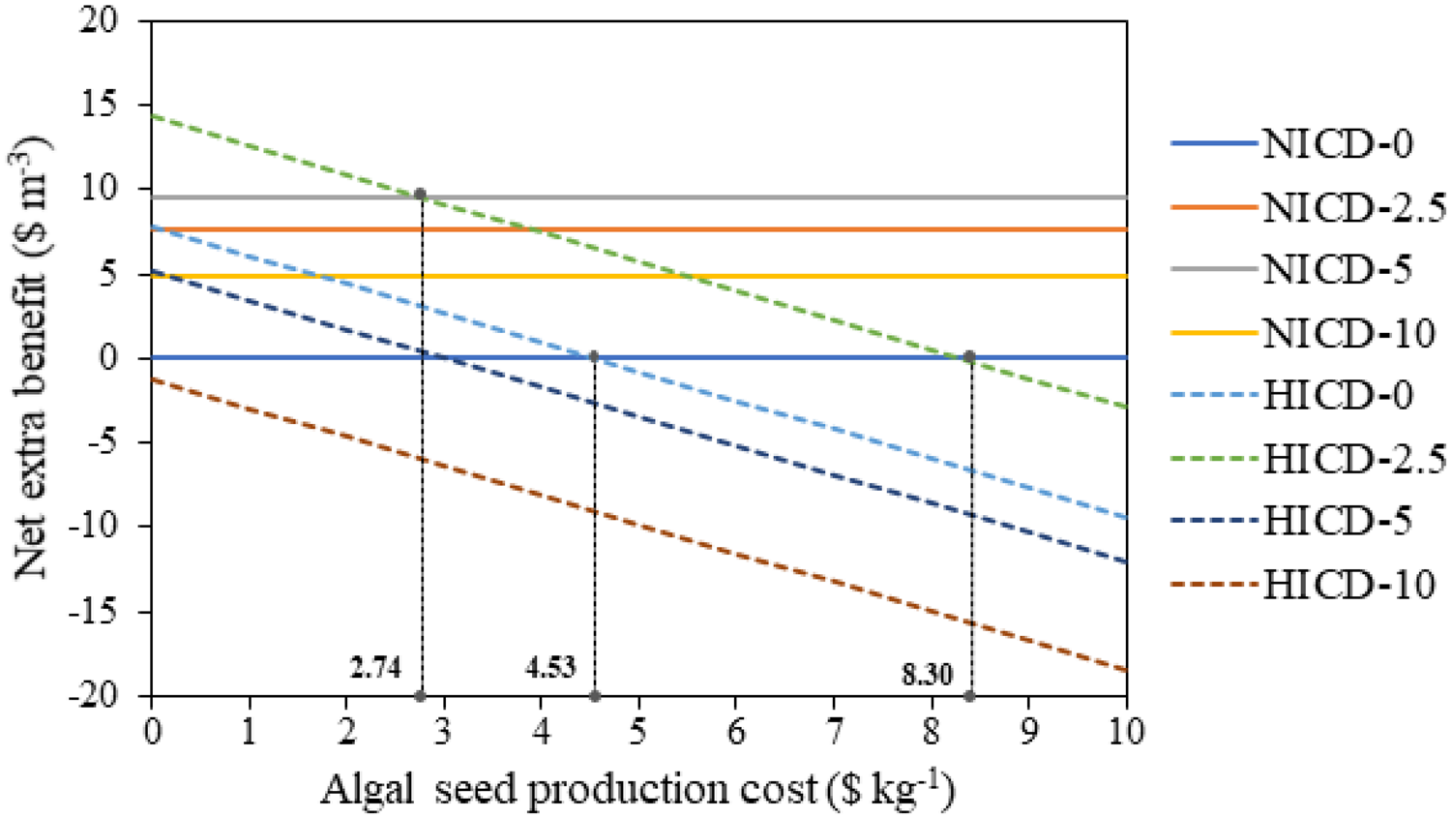
Modelling of the net extra benefit from GABA supply and ICD increase for starch production under nitrogen deprivation as a function of the algal seed production cost in *T. subcordiformis*

**Fig. 8:**
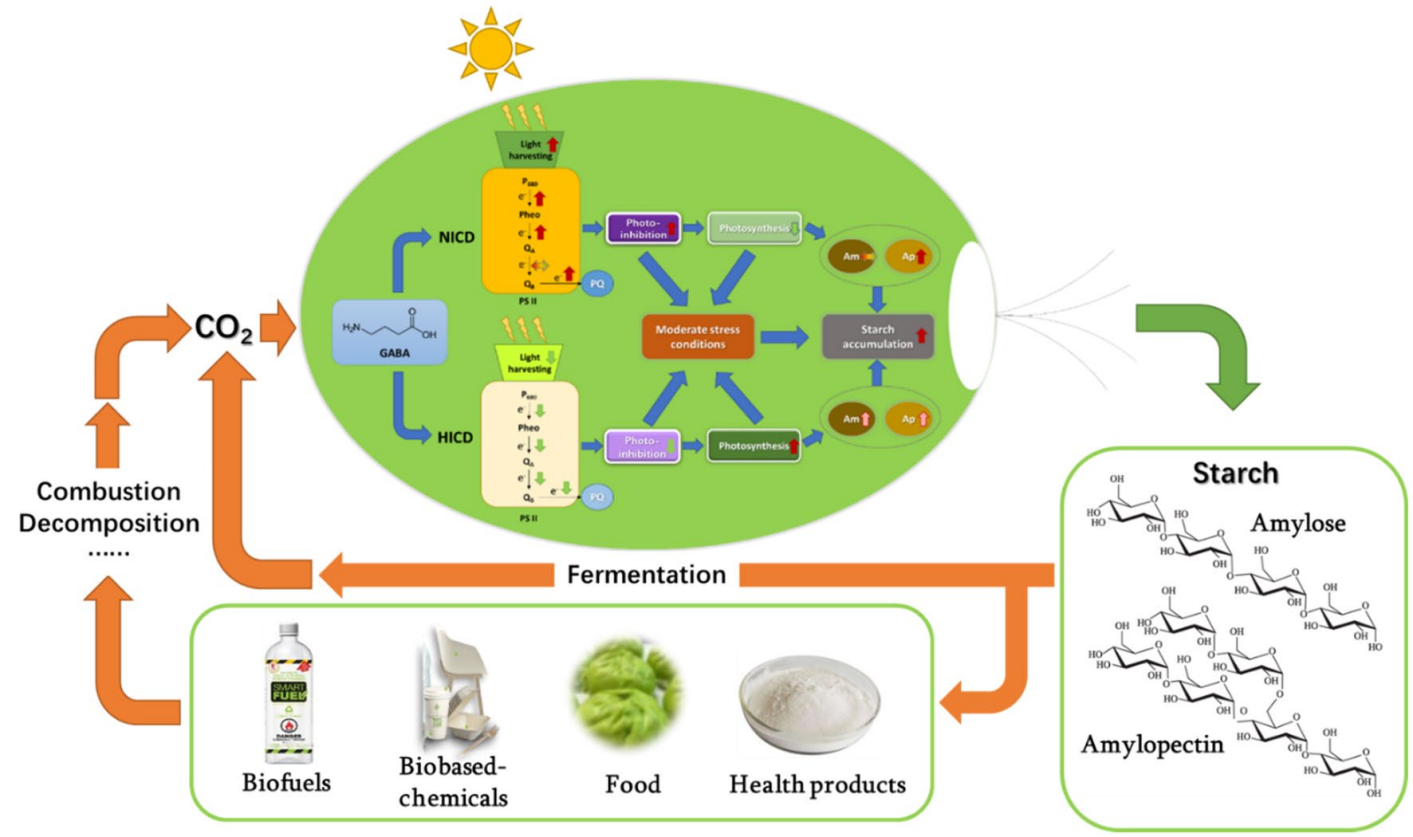
Proposed circulation of CO_2_ to the intermediate molecule starch via microalgae for potentially sustainable biomanufacturing

To further evaluate the net extra benefit, the extra investment involved (increased algal biomass seed and GABA supplied) should be subtracted. The increase of ICD requires more algal seed in the culture, and the cost of algae production for the seed varies depending on cultivation systems and locations (Acién et al. 2014), which could influence the economy of the HICD culture. Therefore, a modelling of the net extra benefit from GABA addition and ICD increase for starch production under nitrogen deprivation as a function of the algal seed production cost in *T. subcordiformis* was performed with a constant GABA cost of 11.5 $ kg^−1^ (Xiong et al. 2017). As shown in Fig. 7, by simply increasing the ICD from NICD (NICD-0) to HICD (HICD-0), a positive net extra benefit could be achieved when the algal seed production cost was lower than 4.53 $ kg^−1^. This compensation point would be largely increased to 8.30 $ kg^−1^ if 2.5 mM GABA was supplied simultaneously (HICD-2.5), indicating that proper GABA addition decreased the demand for restricted biomass production cost, which could enhance the applicability of HICD strategy in a broader range of cultivation systems and locations. In addition, the combined application of HICD and GABA addition would be more economical only if the algal seed production cost was reduced to a certain value. For example, the net extra benefit could become higher by simultaneously increasing ICD and adding 2.5 mM GABA (HICD-2.5) only when the algal seed production cost was decreased to less than 2.74 $ kg^−1^ compared with that under NICD with 5 mM GABA addition (NICD-5, Fig. 7). Currently, the production cost of microalgae biomass can be reduced to ~ 2.71 $ kg^−1^ (Muhammad et al. 2021), which is favourable for the combined HICD-GABA scenario. Under such cost value, the net extra benefit from the ICD increase and GABA addition was calculated. The results (Table 3) showed that under NICD, GABA addition with all the concentrations tested could bring along positive net benefit, with the highest value of 9.59 $ m^−3^ culture obtained under 5 mM GABA addition. The increase of ICD alone (HICD-0) could get net benefit of 3.14 $ m^−3^ culture, and if combined with 2.5 mM GABA addition, the highest net extra benefit of 9.64 $ m^−3^ culture could be achieved (Table 3, HICD-2.5). These analyses indicated that the increase of ICD or/and GABA supply could contribute to the economic production of starch in *T. subcordiformis*. If further taken into the consideration of the probable reduction of harvesting cost with the higher algal biomass obtained under HICD (Shen et al. 2009), the advantage could be more highlighted. Specifically, by adding 2.5 mM GABA under HICD where the best net benefit could be attained, the starch content could exceed 62%DW with a high starch yield of more than 2.5 g L^−1^, which outstood among the majority of the starch-producing microalgae (including *T. subcordiformis* reported previously) cultivated with a batch mode and similar culture conditions under nutrient deprivation (Ran et al. 2019) (Table 2). Although these results must be further validated with larger-scale tests and could only be considered as a projection at present, the strategy presented herein could contribute to the circulation of CO_2_ derived from fermentation (manufacturing stage) and combustion (consumption stage) processes to the intermediate molecule starch via microalgae for potentially economic and sustainable biofuels/bio-based chemicals production as well as for manufacturing food and health products (as discussed in “Starch composition” section, Fig. 8).

## Conclusions

The HICD and/or proper GABA addition facilitated photosynthetic starch production in *T. subcordiformis* under nitrogen deprivation. HICD with 2.5 mM GABA supply almost doubled the starch yield and theoretical productivity along with 22% improvement of starch content compared to those under NICD without GABA addition. GABA exhibited distinct regulatory effects on photosynthetic performance under different ICDs: it enhanced excessive light energy absorption and electron transfer through the reaction centre of PS II that caused intensified photoinhibition under NICD, while it protected the photosynthesis in a reverse way under HICD, both of which enabled a suitable stress status along with a sufficient photosynthetic activity that benefited starch accumulation. The HICD and/or GABA supply changed the starch composition, with particularly amylopectin accumulation boosted, which was suitable for fermentation-based biofuels production and biomaterials manufacturing as well as being applied in food technology and other chemical industries. Net extra benefit could be obtained from ICD increase and/or proper GABA addition for starch production in *T. subcordiformis*. The application of the combined HICD-GABA supply strategy could contribute to the economic and sustainable production of starch from CO_2_ by microalgae, which constituted a circular paradigm for biomanufacturing.

### Supplementary Information

**Additional file 1: Table S1.** Effect of exogenous GABA supply on protein content (%DW) in *T. subcordiformis *exposed to nitrogen deprivation under NICD and HICD conditions. **Figure S1.** Correlation analysis of stress status (*F*_v_/*F*_m_) and relative amylopectin (Ap) production (Ap theoretical productivity/total starch theoretical productivity) in *T. subcordiformis* exposed to nitrogen deprivation under NICD and HICD conditions with GABA supply on Day 4.

## Data Availability

All data generated or analysed during this study are included in this published article.
