# Supplementary material for: Fine-tuned regulation of photosynthetic performance via γ-aminobutyric acid (GABA) supply coupled with high initial cell density culture for economic starch production in microalgae
Source: Bioresour Bioprocess. 2022 May 12;9(1):52. doi: 10.1186/s40643-022-00541-3 (PMC10992858; doi:10.1186/s40643-022-00541-3)
Supplement: Supplementary file 1 — Additional file 1: Table S1. Effect of exogenous GABA supply on protein content (%DW) in T. subcordiformis exposed to nitrogen deprivation under NICD and HICD conditions. Figure S1. Correlation analysis of stress status (Fv/Fm) and relative amylopectin (Ap) production (Ap theoretical productivity/total starch theoretical productivity) in T. subcordiformis exposed to nitrogen deprivation under NICD and HICD conditions with GABA supply on Day 4. [file 40643_2022_541_MOESM1_ESM.docx]

| ICD | GABA (mM) | Protein content (%DW) | | |
| --- | --- | --- | --- | --- |
|  |  | Day 0 | Day 2 | Day 4 |
| NICD | 0 | 27.41 | 17.47±3.09 | 13.18±3.74 |
|  | 2.5 |  | 17.86±3.23 | 12.62±2.43 |
|  | 5 |  | 17.99±4.77 | 12.56±0.33 |
|  | 10 |  | 13.08±1.24 | 12.64±0.53 |
| HICD | 0 | 23.51 | 13.17±0.61 | 14.52±1.41 |
|  | 2.5 |  | 11.14±0.29 | 15.27±1.63 |
|  | 5 |  | 15.11±2.75 | 15.60±1.41 |
|  | 10 |  | 14.60±2.75 | 15.09±0.52 |

**Table S1** Effect of exogenous GABA supply on protein content (%DW) in *T. subcordiformis* exposed to nitrogen deprivation under NICD and HICD conditions.


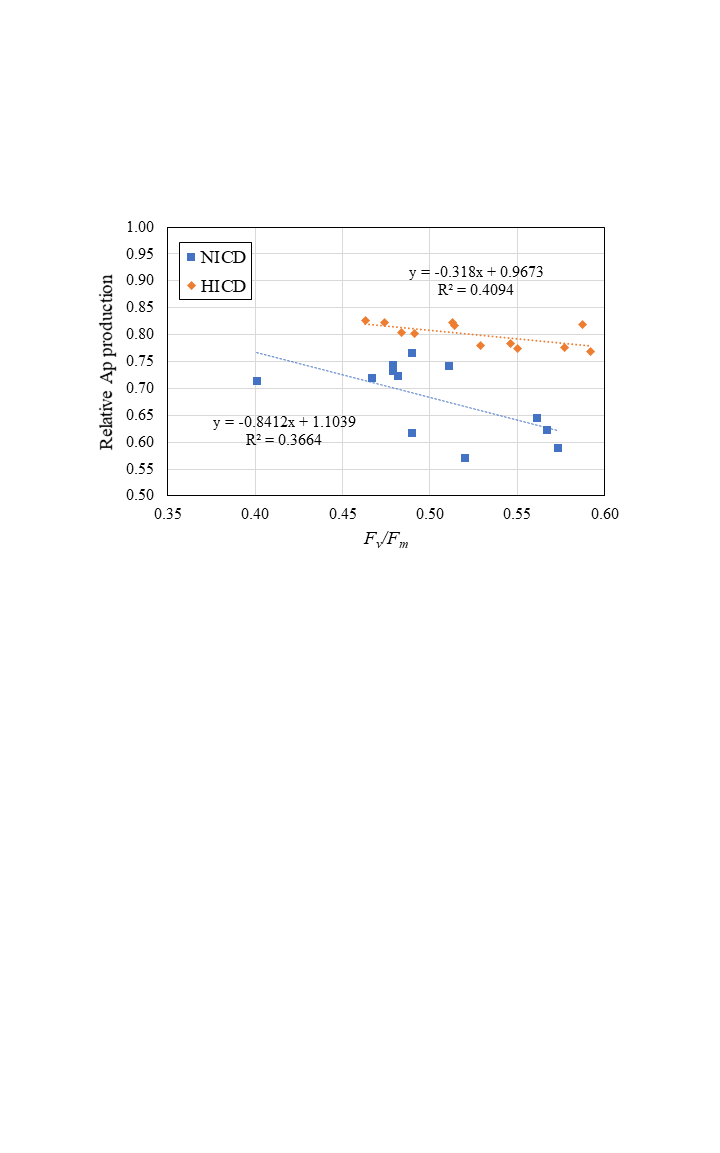


**Fig. S1.** Correlation analysis of stress status (*F_v_/F_m_*) and relative amylopectin (Ap) production (Ap theoretical productivity/total starch theoretical productivity) in *T. subcordiformis* exposed to nitrogen deprivation under NICD and HICD conditions with GABA supply on Day 4.
